# Immune microenvironment in patients with mismatch‐repair‐proficient oligometastatic colorectal cancer exposed to chemotherapy: the randomized MIROX GERCOR cohort study

**DOI:** 10.1002/1878-0261.13173

**Published:** 2022-02-09

**Authors:** Marine Jary, Wen‐Wei Liu, Dongyao Yan, Isaac Bai, Andrea Muranyi, Elise Colle, Isabelle Brocheriou, Anthony Turpin, Nina Radosevic‐Robin, Pierre Bourgoin, Frédérique Penault‐Llorca, Romain Cohen, Dewi Vernerey, Thierry André, Christophe Borg, Kandavel Shanmugam, Magali Svrcek

**Affiliations:** ^1^ Multidisciplinary Group in Oncology (GERCOR) Paris France; ^2^ Department of Surgical and Medical Oncology University Hospital of Clermont‐Ferrand France; ^3^ Ventana Medical Systems Inc. Tucson AZ USA; ^4^ Department of Medical Oncology University Hospital of Beaujon Clichy France; ^5^ Department of Pathology Assistance Publique‐Hôpitaux de Paris Pitié‐Salpêtrière Hospital Sorbonne University Paris France; ^6^ Department of Medical Oncology University Hospital of Lille France; ^7^ Department of Pathology, Centre Jean Perrin Clermont‐Ferrand France; ^8^ University Clermont Auvergne INSERM U1240 (IMoST - Molecular Imaging & Theranostic Strategies) Clermont‐Ferrand France; ^9^ Department of Pathology Assistance Publique‐Hôpitaux de Paris Saint‐Antoine Hospital Sorbonne University Paris France; ^10^ Department of Medical Oncology Assistance Publique‐Hôpitaux de Paris Saint‐Antoine Hospital Sorbonne University Paris France; ^11^ Methodology and Quality of Life in Oncology Unit Besançon University Hospital France; ^12^ INSERM EFS BFC UMR1098, RIGHT, Interactions Hôte‐Greffon‐Tumeur/Ingénierie Cellulaire et Génique University of Bourgogne Franche‐Comté Besançon France; ^13^ Department of Medical Oncology University Hospital of Besançon France

**Keywords:** immune profile, oligometastatic colorectal cancer, PD‐L1, pMMR, T lymphocytes

## Abstract

In the era of immune checkpoint inhibitors, understanding the metastatic microenvironment of proficient mismatch repair/microsatellite stable (pMMR/MSS) colorectal cancer (CRC) is of paramount importance to both prognostication and the development of more effective novel therapies. In this study, primary and paired metastasis tissue samples were collected from patients with resectable metastatic CRC treated with adjuvant FOLFOX or peri‐operative chemotherapy in the MIROX phase III prospective study. In total, 74 cancer tissues were stained for CD3, CD8, Forkhead box protein 3 (FOXP3), programmed cell death protein‐1 (PD‐1, invasive front, stromal, intra‐epithelial compartments), and programmed death‐ligand 1 (PD‐L1, tumor, immune cells). The immune profiling of primary CRC had a limited value to predict the immune context of paired metastases for all markers but CD3+. The expression of CD8 and PD‐L1 was higher in metastases after neoadjuvant FOLFOX. In metastases, both CD3 T cells at the invasive front and PD‐L1 expressions on immune cells were predictive of better disease‐free survival. These results show that the effect of FOLFOX on modifying the immune microenvironment in resected CRC metastases and measurement of PD‐L1 expression and tumor‐infiltrating CD8 T cells in pMMR/MSS metastatic tissue samples could improve treatment strategies of metastatic CRC patients.

## Graphical abstract

Here, we characterized the immune microenvironment of proficient mismatch repair/microsatellite stable oligometastatic patients with colorectal cancer (CRC) treated with the neoadjuvant FOLFOX. Primary tumor immune profiling had limited predictive value in estimating the metastatic immune context. CD3 T cells and PD‐L1 immune cells at the invasive front were predictive of disease‐free survival. In addition, the expression of CD8 and PD‐L1 was higher after FOLFOX treatment. Thus, CD8^high^/PD‐L1^high^ signature could be related to chemotherapy response and could improve treatment strategies of metastatic patients with CRC.

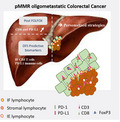

**Abbreviations**
CC1 buffer: cell conditioning 1 buffer
CEA: carcinoembryonic antigen
CI: confidence interval
CMS: consensus molecular subtype
CRC: colorectal cancer
DFS: disease‐free survival
dMMR/MSI: deficient mismatch repair /microsatellite instable
FFPE: formalin‐fixed, paraffin‐embedded
FOXP3: forkhead box protein 3
GERCOR: Groupe Coopérateur Multidisciplinaire en Oncologie
HR: hazard ratios
ICIs: immune checkpoint inhibitors
IE: intra‐epithelial
IF: invasive front
IFN‐γ: interferon gamma
IHC: immunohistochemistry
mCRC: metastatic CRC
MDSC: myeloid derived suppressor cell
MEK: mitogen‐activated protein kinase kinase
MHC: major histocompatibility complex
MLH1: MutL homolog 1
MSH2: MutS homolog 2
MSH6: MutS homolog 6
omCRC: oligometastatic CRC
PD‐1: programmed cell death protein‐1
PD‐L1: programmed death ligand 1
pMMR/MSS: proficient mismatch repair/microsatellite stable
PMS2: PMS1 homolog 2
TRG: tumor regression grade

## 1 Introduction

Accumulating evidence suggests that the adaptive immune system can influence cancer progression and that the quantification of tumor‐infiltrating lymphocytes may improve prognostic ability of the staging system in patients with solid tumors. In colorectal cancer (CRC), the impact of immune cell infiltration in the primary tumor on survival has been demonstrated [1, 2]. Patients with metastatic CRC (mCRC) in the liver have heterogeneous clinical outcomes. Indeed, 70% of patients with curatively resected metastases will relapse and half of these will ultimately die [3, 4]. Clinico‐pathological prognostic factors like the tumor regression grade (TRG) have been proposed to identify patients who may be at risk for recurrence [5], but none of these markers has been sufficiently informative to correctly predict the outcome. In the era of personalized medicine, an identification of prognostic and predictive biomarkers is essential. Regarding mCRC, the immune microenvironment of the liver metastases reflects an important aspect of the overall portrait of the patient's disease, especially the heterogeneity compared to the primary tumor and its clinical impact [6, 7, 8, 9]. The immune microenvironment of colorectal metastases has not been fully investigated, and published studies are often limited to CD4, CD8, and regulatory T cells [10]. In addition, several chemotherapy regimens such as oxaliplatin seem to have a preponderant role in antitumor immunologic infiltration, with a stimulating effect on the peritumoral immune response [11, 12]. In particular, immunogenic cell death is provoked by FOLFOX and accompanied by tumor‐targeting immune responses, release of damage‐associated molecular patterns, and recruitment of antigen‐presenting immune cells. Interestingly, the EORTC phase III study 40 983 of 82 patients with resected colorectal liver metastases (38 in the surgery with peri‐operative FOLFOX chemotherapy arm and 44 in the surgery alone arm) [13] showed for the first time that chemotherapy influences immune cell profiles, independent of patient characteristics. In this latter study, abundance of CD3 T‐cell lymphocytes at the invasive margin of the resected metastasis specimens appeared to be prognostic. Moreover, immune infiltration of lymphocytes was associated with increased progression‐free survival.

The efficacy of immune checkpoint inhibitors (ICIs) in mismatch repair deficient (dMMR)/microsatellite instable (MSI) mCRC is now well established [14, 15, 16, 17, 18]. However, questions remain regarding the role of ICIs for the treatment of MMR‐proficient (pMMR)/microsatellite stable (MSS) mCRC. The combination of ICIs with other anticancer drugs is currently being evaluated in pMMR/MSS mCRC. The disappointing results of the phase III IMblaze 370 trial (atezolizumab with or without cobimetinib *versus* regorafenib) raise concerns regarding the testing ICI‐based strategies without decision‐guiding biomarkers in pMMR/MSS mCRC. Contrarily, the NICHE study provided hypothesis‐generating data for patients with localized pMMR/MSS colon cancer [19]. Thus, deeper understanding of the metastatic microenvironment of pMMR/MSS mCRC and particularly oligometastatic CRC (omCRC) could improve selection of patients who may benefit from ICI combinations and other immunogenic drugs such as oxaliplatin.

Programmed death‐ligand 1 (PD‐L1) is expressed by tumor cells and certain immune cell types (dendritic cells, macrophages, B lymphocytes, Natural Killer cells). T cells expressing programmed cell death protein‐1 (PD‐1) exhibit suppressed proliferation through PD‐1/PD‐L1 interaction. However, PD‐1 expression and PD‐L1 expression in primary CRC are associated with favorable outcomes [20, 21]. In most cancers treated with anti‐PD‐1 or anti‐PD‐L1 antibodies, the response rate is often higher in tumors expressing higher levels of PD‐L1‐positive immune cells. PD‐L1‐expressing tumor cells have been shown to regulate host immunity in the CRC microenvironment [22]. In the pMMR cohort of the NICHE study, the presence of T cells with co‐expression of CD8 and PD‐1 was the only biomarker found to predict major or partial pathological response [19]. Data regarding the expression of PD‐L1 in CRC liver metastases and notably the interactions of PD‐L1 with elements of the immune tumor microenvironment, as well as patient outcome, have recently been described [23]. No correlation between tumor‐specific PD‐L1 expression and survival was shown, confirming the results from other studies [24]. However, contradictory results are observed between the metastatic and early‐stage settings [25], and few study yet assessed the expression of PD‐L1 in a homogeneous cohort of matched primary and metastatic pMMR/MSS omCRC [26].

In this study, we aimed to extensively characterize the immune microenvironment in patients with resectable mCRC treated or not with neoadjuvant FOLFOX to highlight an immunologic signature in this setting. We described potential tumor heterogeneity in chemo‐naive patients with synchronous metastases and investigated if the immune infiltrate in resected colorectal metastases could be predictive of survival. The influence of FOLFOX‐based chemotherapy and PD‐1 and PD‐L1 expression on the immune microenvironment and patient survival was further investigated.

## 2 Materials and methods

### 2.1 Study population

In total, 74 mCRC patients with available tissues from both primary tumors and paired metastases out of 284 included in the open‐label prospective phase III MIROX trial were analyzed in this analysis. Patients with resectable or resected synchronous or metachronous metastases (only one site in liver, lung, ovary, or peritoneum) were treated with six cycles of FOLFOX4 (oxaliplatin 85 mg·m^−2^) or FOLFOX7 (oxaliplatin 130 mg·m^−2^) before metastasis resection followed by adjuvant chemotherapy (FOLFOX or FOLFIRI). The dose of oxaliplatin was randomly assigned at the beginning of the study [27]. The primary CRC was resected before diagnosis of metastasis and neoadjuvant chemotherapy. This trial was approved by the local Ethics Committees at participating GERCOR (*Groupe Coopérateur Multidisciplinaire en Oncologie*) centers. All patients provided their written informed consent to receive treatment and participate in translational analysis.

Formalin‐fixed, paraffin‐embedded (FFPE) primary CRC tumor specimens were obtained prior to chemotherapy. Paired metastatic lesions were collected prior to adjuvant chemotherapy or after neoadjuvant chemotherapy and centralized at the Department of Pathology, Saint‐Antoine Hospital (Paris, France), constituting the study cohort, BIOMIROX. Pathological response of CRC liver metastasis in patients treated with peri‐operative chemotherapy was estimated by the TRG pathological response score [5]. The study methodologies conformed to the standards set by the Declaration of Helsinki.

### 2.2 Immunohistochemistry analysis

Immunohistochemistry (IHC) staining was performed on serial sections from surgically resected specimens (VENTANA BenchMark ULTRA automated staining instrument at Pitié‐Salpêtrière Hospital). Briefly, FFPE tissue sections were deparaffinized, pretreated with Cell Conditioning 1 for antigen retrieval, and treated to inactivate the endogenous peroxidase and then incubated with CONFIRM anti‐CD3 (2GV6) rabbit monoclonal antibody, anti‐CD8 (SP239) rabbit monoclonal antibody, anti‐FOXP3 (SP97) rabbit monoclonal antibody, anti‐PD‐1 (NAT105) mouse monoclonal antibody, and anti‐PD‐L1 (SP263) rabbit monoclonal antibody. Staining was visualized using the OptiView DAB IHC Detection Kit (Ventana Medical System, Inc., Tucson, AZ, USA). Following detection, all slides were counterstained with hematoxylin II and bluing reagent for 4 min each, and coverslips were applied.

The density of tumor‐infiltrating immune CD3 and CD8 T cells was semiquantitatively scored at the invasive front (IF; cells localized in stroma adjacent to the invasive tumor margin) and the intratumoral (or stromal) compartment [28] and graded as follows: 0, no positive cells; 1, scattered positive cells; 2, moderate number of positive cells; 3, abundant occurrence of positive cells. For CD3 T cells, the intra‐epithelial (IE) compartment was also assessed. PD‐1 and FOXP3 were scored semiquantitatively, both at the IF and stromal compartment, as follows: 0, no positive cell or scattered cells; 1, moderate number of positive cells, and 2, numerous positive cells. Positive PD‐L1 expression was defined as any staining ≥ 1%, in either infiltrating inflammatory cells or membranous‐site tumor cells [29]. The percentage of cells demonstrating PD‐L1 staining was scored in 5% increments, and high PD‐L1 level was defined as ≥ 5%, based on published literature [30, 31, 32, 33, 34]. Tissue samples were evaluated by an experienced pathologist (MS) blinded to clinical information, treatment regimens, and outcomes (Table S1).

Tumor tissue sections were double‐stained for PD‐L1 and CD8 using a fully automated procedure in a Benchmark Ultra automate (Ventana/Roche, Tucson, AZ, USA). Epitope retrieval was performed in Cell Conditioning 1 (CC1 buffer) for 60 min at 95 °C. PD‐L1 was detected by Ventana PD‐L1 SP263 Assay (Roche Diagnostics, Meylan, France), per manufacturer's instructions. CD8 was detected by clone SP239 (Abcam, Cambridge, UK) at 1/100 for 60 min at room temperature. For PD‐L1, the antigen‐antibody reaction was revealed by OptiView DAB IHC Detection Kit and, for CD8 by UltraView Universal AP Red Detection Kit (red signal), both from Roche Diagnostics. Co‐staining was appreciated by a semiquantitative method.

The expression of MutL homolog 1 (MLH1, dilution 1/70, clone G168‐728; Pharmingen, San Diego, CA, USA), MutS homolog 2 (MSH2, dilution 1/100, clone FE11, Calbiochem, Oncogene Research Products, Cambridge, MA, USA), MutS homolog 6 (MSH6, dilution 1/100, clone 44; Becton Dickinson, Lexington, NC, USA), and PMS1 homolog 2 (PMS2, clone A16‐4, 1 : 150 dilution; BD PharMingen, Le Pont‐de‐Claix, France) was assessed. Immunostaining of MLH1, MSH2, MSH6, and PMS2 in tumor cells was evaluated as positive [mismatch repair (MMR)‐proficient (pMMR)] or negative [MMR‐deficient (dMMR)]. Tumors were considered negative when there was a complete absence of nuclear staining of neoplastic cells in the presence of an internal positive control.

### 2.3 Statistical analysis

All IHC markers but PD‐L1 were semiquantitatively assessed. Therefore, they were treated as categorical variables in this study. The Cox proportional hazards model was used to estimate hazard ratios (HRs) and *P* values with disease‐free survival (DFS). The association between clinical, biomarker parameters, and survival was estimated with univariate Cox proportional hazards models and HR with 95% confidence interval (CI) were given. Multivariate Cox models were investigated including clinico‐biological parameters with *P* value < 0.05 in univariate analysis. *P* value < 0.05 was considered statistically significant. Spearman correlations between metastatic immune infiltrate and TRG scoring were estimated. Analyses were performed by sas version 9.4 (SAS Institute Inc., Cary, NC, USA).

## 3 Results

### 3.1 Study cohort characteristics

A total of 74 patients with pMMR CRC were included in the current study. All baseline characteristics are summarized in Table S2 and in the flowchart (Fig. S1). The median age of patients was 61 years (range 29–75). Forty‐one patients had nearby lymph node metastases of the primary, and 82.6% were left‐sided tumors (including rectum). Most patients (*n* = 65) had liver‐only metastases, and nine had another only one site of distant metastases [lung (*n* = 3), ovary (*n* = 1), peritoneal location (*n* = 5)]. The median number of metastases was 2 (range, 1–7). Twenty‐four patients had metachronous metastases. The mean preoperative carcinoembryonic antigen (CEA) level was 55.9 ng·mL^−1^ (*n* = 70). Out of the 74 patients analyzed, 34 received adjuvant chemotherapy after surgery of their metastases (‘chemo‐naïve’ patients). Among patients treated with neoadjuvant chemotherapy, two did not receive oxaliplatin.

### 3.2 Immune profiling between the primary CRC and matched metastasis

**Fig. 1:**
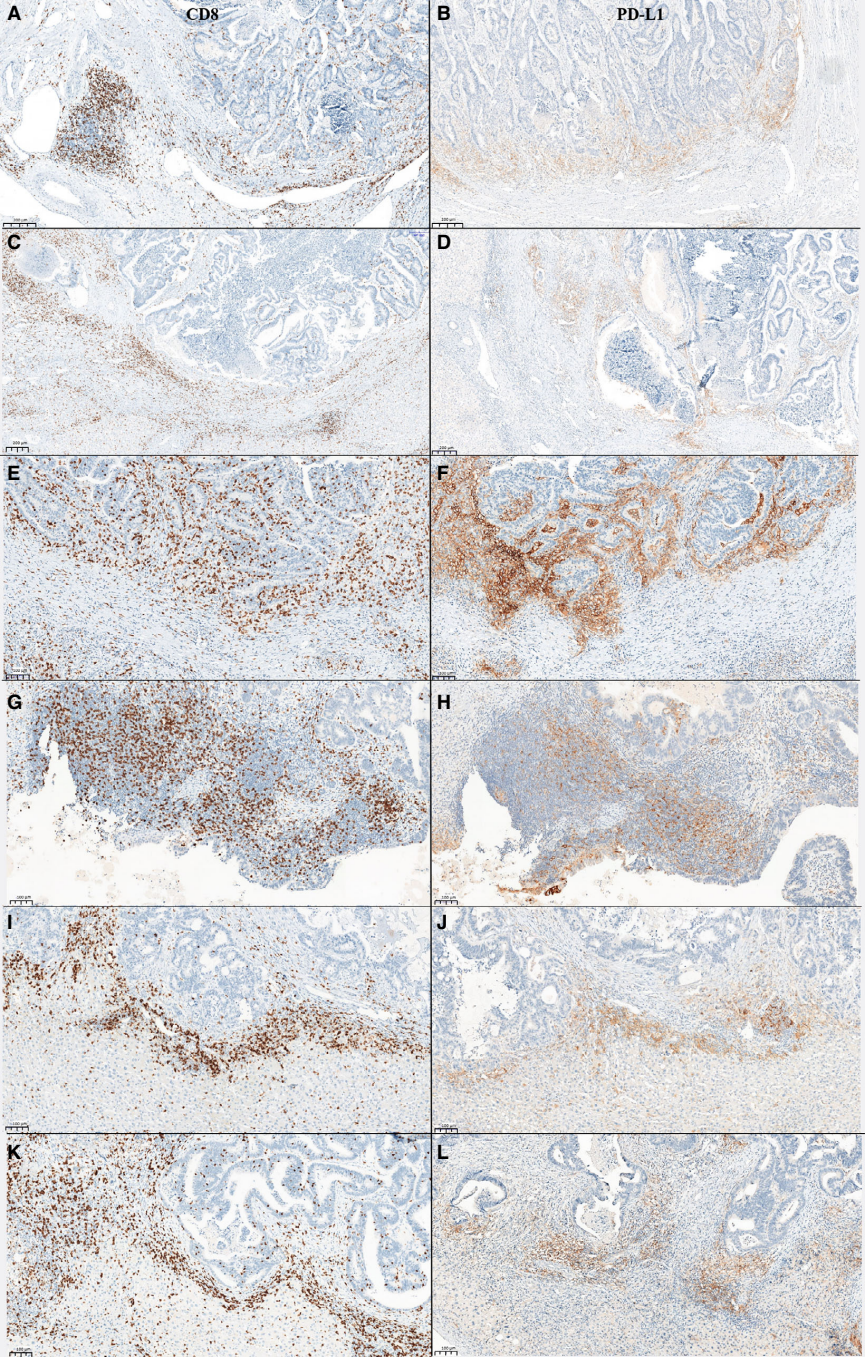
Representative images of immunohistochemistry staining of CD8 and PD‐L1. (A) High CD8 expression in the invasive front of the primary tumor, (B) high PD‐L1 expression in the invasive front of the primary tumor of the same patient, (C) high CD8 expression on immune cell in the invasive front of the liver metastasis of the same patient; (D) high PD‐L1 expression on immune cell in the invasive front of the liver metastasis of the same patient, (E) high CD8 expression in the invasive front of a liver metastasis of a second patient, (F) high PD‐L1 expression in the invasive front of a liver metastasis of the same second patient, (G) high CD8 expression in the invasive front of an ovary metastasis of a third patient, (H) high PD‐L1 expression in the invasive front of an ovary metastasis of the same third patient, (I) high CD8 expression in the invasive front of a liver metastasis of a fourth patient, (J) high PD‐L1 expression in the invasive front of a liver metastasis of the same fourth patient, (K) high CD8 expression in the invasive front of a liver metastasis of a fifth patient, (L) high PD‐L1 expression in the invasive front of a liver metastasis of the same fifth patient. Scale bar for A–D = 200 µm. Scale bar for E–L = 100 µm.

The distribution of semiquantitative scores of CD3, CD8, FOXP3, PD‐1, and PD‐L1 (Fig. 1, Table 1, Table S1, Fig. S2) showed considerable heterogeneity, both in primary tumors and metastases, in chemo‐naïve patients and those treated with neoadjuvant chemotherapy before surgery. Infiltration of CD3 T lymphocytes was the strongest in stroma and the IF both in primary tumors and metastases.

**Table 1:** Distribution of biomarkers in the primary tumor and metastatic sites.

	IHC	CD3 IF (%)	CD3 stroma (%)	CD3 IE (%)	CD8 IF (%)	CD8 stroma (%)	PD‐1 IF (%)	PD‐1 stroma (%)	Foxp3 IF (%)	Foxp3 stroma (%)		PD‐L1 tumoral cells (%)	PD‐L1 immune cells (%)
Primary tumors													
All (*N* = 74)	IHC 3+	11 (14.9)	15 (20.5)	14 (19.2)	0	0	NA	NA	NA	NA			
	IHC 2+ and 3+	33 (44.6)	42 (57.5)	26 (35.6)	6 (8.5)	8 (11.3)	2 (2.7)	1 (1.4)	3 (4.1)	11 (14.9)	≥ 5	5 (6.8)	42 (57.5)
	IHC 0+ and 1+	41 (55.4)	31 (42.5)	47 (64.4)	65 (91.5)	63 (88.7)	71 (97.3)	73 (98.6)	71 (95.9)	63 (85.1)	< 5	68 (93.2)	31 (42.5)
Associated with neoadjuvant metastases (*N* = 40)	IHC 3+	4 (10.0)	8 (20.0)	7 (17.5)	0	0	NA	NA	NA	NA			
	IHC 2+ and 3+	16 (40.0)	22 (55.0)	13 (32.5)	2 (5.3)	5 (13.2)	1 (2.5)	0	2 (5.0)	4 (10.0)	≥ 5	2 (5.1)	22 (56.4)
	IHC 0+ and 1+	24 (60.0)	18 (45.0)	27 (67.5)	36 (94.7)	33 (86.8)	39 (97.5)	40 (100)	38 (95.0)	36 (90.0)	< 5	37 (94.9)	17 (43.6)
Associated with chemo‐naive metastases (*N* = 34)	IHC 3+	7 (20.6)	7 (21.2)	7 (21.2)	0	0	NA	NA	NA	NA			
	IHC 2+ and 3+	17 (50.0)	20 (60.6)	13 (39.4)	4 (12.1)	3 (9.1)	1 (3.0)	1 (2.9)	1 (2.9)	7 (20.6)	≥ 5	3 (8.8)	20 (58.8)
	IHC 0+ and 1+	17 (50.0)	13 (39.4)	20 (60.6)	29 (87.9)	30 (90.9)	32 (97)	33 (97.1)	33 (97.1)	27 (79.4)	< 5	31 (91.2)	14 (41.2)
Metastases													
All (*N* = 74)	IHC 3+	16 (21.9)	10 (13.5)	12 (16.4)	2 (2.8)	3 (4.2)	NA	NA	NA	NA			
	IHC 2+ and 3+	63 (86.3)	30 (40.5)	20 (27.4)	23 (31.9)	16 (22.2)	9 (12.7)	4 (5.6)	0	4 (5.6)	≥ 5	7 (9.7)	44 (61.1)
	IHC 0+ and 1+	10 (13.7)	44 (59.5)	53 (72.6)	49 (68.1)	56 (77.8)	62 (87.3)	67 (94.4)	72 (100)	68 (94.4)	< 5	65 (90.3)	28 (38.9)
Neoadjuvant (*N* = 40)	IHC 3+	11 (28.2)	7 (17.5)	5 (12.5)	1 (2.6)	2 (5.1)	NA	NA	NA	NA			
	IHC 2+ and 3+	32 (82.1)	19 (47.5)	8 (20.0)	16 (41.0)	10 (25.6)	6 (15.8)	3 (7.9)	0	0	≥ 5	5 (13.2)	21 (55.3)
	IHC 0+ and 1+	7 (17.9)	21 (52.5)	32 (80.0)	23 (59.0)	29 (74.4)	32 (84.2)	35 (92.1)	39 (100)	39 (100)	< 5	33 (86.8)	17 (44.7)
Chemo‐naive (*N* = 34)	IHC 3+	5 (14.7)	3 (8.8)	7 (21.2)	1 (3.0)	1 (3.0)	NA	NA	NA	NA			
	IHC 2+ and 3+	31 (91.2)	11 (32.4)	12 (36.4)	7 (21.2)	6 (18.2)	3 (9.1)	1 (3.0)	0	4 (12.1)	≥ 5	2 (5.9)	23 (67.6)
	IHC 0+ and 1+	3 (8.8)	23 (37.6)	21 (63.6)	26 (78.8)	27 (81.8)	30 (90.9)	32 (97.0)	33 (100)	29 (87.9)	< 5	32 (94.1)	11 (32.4)

To avoid the potential confounding effect of chemotherapy on immune cell infiltration, we further examined the distribution scores by restricting the analyses to 34 chemo‐naïve patients only (Fig. S2). The vast majority of these patients (*n* = 31) demonstrated high density of CD3 T cells (IHC score 2–3) in the IF of metastatic sites [liver (*n* = 25), lung (*n* = 2), ovary (*n* = 1), peritoneal (*n* = 3)]. Seventeen patients in the chemo‐naïve cohort had a high expression of CD3 cells (IHC 2 and 3) in the IF of the primary tumor (Table 1). Interestingly, this expression was strongly correlated (16 out of 17 patients, 94%) with that observed in the metastases compared, but it was not the case for stromal and intraepithelial compartments CD3 cells (IHC 2 and 3) of the primary tumor and their matched metastases (Table 1 and Table S1).

PD‐1 overexpression on CD8 T cells is known as an exhaustion biomarker, but it also reflects antigen‐experienced lymphocytes. Conversely, PD‐L1 expression on tumor or immune cells is induced by an interferon‐mediated signaling. We characterized therefore CD3 and CD8 expression according to PD‐L1 expression in the pMMR/MSS mCRC study cohort. A high level of PD‐L1 expression was observed predominantly in immune cells [20 (58.8%) vs 3 (8.8%) in tumoral cells; Table S1] in CRC. Among the 33 patients for whom CD8 staining was available, four had high CD8 immune infiltrate (IHC scores 2–3) in the IF of the primary tumor (Table S1). Three of the latter patients had a PD‐L1 expression ≥ 5% in immune cells (CD8^high^/PD‐L1^high^ patients; Fig. 1). Of the 29 patients with low CD8 score, 17 showed high PD‐L1 expression (CD8^low^/PD‐L1^high^ patients; Table 1 and Table S1). By comparison, 26 patients had low CD8 immune infiltrate in the IF of metastases, 10 of whom were classified as PD‐L1^low^ (< 5%; CD8^low^/PD‐L1^low^). Seven patients had high CD8 T‐cell infiltration in the IF. In all these patients, a high PD‐L1 score (≥ 5% of expression by immune cells; Fig. 1, Table 1, and Table S1) was observed. In order to better appreciate the colocalization of CD8 and PD‐L1, their costaining was performed in six of these seven patients. The colocalization of CD8 and PD‐L1 was observed in ≥ 20% of inflammatory cells. In four out of seven cases, a colocalization was detected in over 50% of cells. Negative controls had no more than 10% of colocalization detected (Fig. S3).

Regulatory T cells in CRC have been described in the noninflamed tumors because of their tolerance properties, but they also play a role for effector T cells, which is to maintain immune homeostasis, even when the antitumor immune response is active [35, 36, 37]. In our study, half of the CD8^high^/PD‐L1^high^ patients had high immune IF FOXP3 infiltration.

Contrary to CD3 staining, the analysis showed that CD8 and PD‐L1 expressions were slightly less correlated between the primary tumors and matched metastases. Fifteen of the 20 patients with a high PD‐L1 expression (i.e., at least 5% of positive immune or tumoral cells) in the primary tumor had also high PD‐L1 expression in the matched metastasis (Table 1 and Table S1). Three out of four patients with high CD8 expression in the primary tumor had also more CD8 T‐cell infiltration in their metastasis. Three patients showing positive PD‐L1 staining in the primary tumor were negative/low for PD‐L1 in the matched metastasis but showed a high CD3 immune infiltration (IHC score 2–3) in the IF of the metastatic tumor.

The above data suggested that the immune profiling performed on the primary tumor has a limited predictive value to estimate the immune context of metastasis. Further analysis focused on the tumor‐infiltrating immune cell in metastasis.

### 3.3 Patients' characteristics according to the expression of IF CD3, IF CD8, and immune PD‐L1 in metastases of chemo‐naive patients

The clinical characteristics of 34 chemo‐naive patients according to the expression of IF CD3, IF CD8, and immune cells PD‐L1 in metastatic tumors are described in Table 2. Most of patients with high IF CD3 expression in metastatic tumors [out of 31 (96.7%); IHC score 2+ and 3+] and all patients with and CD8+ and PD‐L1 in metastases (*n* = 7) had tumors less than 5 cm in diameter and 70% (21 out of 31 patients) and 85%, respectively, had only one metastatic site. The CEA level before metastases resection was low in these patients.

**Table 2:** Patients' characteristics according to invasive front CD3, invasive front CD8, and immune cell PD‐L1 expressions in chemo‐naive patients with metastases.

Parameter	Invasive front CD3 in metastases			Invasive front CD8 and immune cells PD‐L1 in metastases			
	CD3 high	CD3 low	*P*	CD8high PD‐L1 high	CD8 low PD‐L1 high	CD8 low PD‐L1 low	*P*
Age (years)							
*N*	31	3		7	16	10	
Median (range)	61 (45–75)	68 (66–74)	0.089	64.2 (45–75)	59.6 (50–71)	64.6 (53–75)	0.332
Longest diameter of metastases (cm), *N* (%)							
*N*	30	3		7	16	9	
≤ 5	29 (96.7)	2 (66.7)	0.176	7 (100.0)	16 (100.0)	7 (77.8)	0.115
> 5	1 (3.3)	1 (33.3)		0 (0.0)	0 (0.0)	2 (22.2)	
N‐stage, *N* (%)							
*N*	30	3		7	16	9	
N0	17 (56.7)	1 (33.3)	0.579	4 (57.1)	11 (68.8)	3 (33.3)	0.247
N+	13 (43.3)	2 (66.7)		3 (42.9)	5 (31.3)	6 (66.7)	
Number of metastases, *N* (%)							
*N*	30	3		7	16	9	
Mean (SD)	1.4 (0.77)	3.3 (3.21)	0.153	1.1 (0.38)	1.6 (0.73)	2.1 (2.09)	0.408
≤ 1	21 (70.0)	1 (33.3)	0.252	6 (85.7)	9 (56.3)	6 (66.7)	0.439
> 1	9 (30.0)	2 (66.7)		1 (14.3)	7 (43.8)	3 (33.3)	
Preoperative CEA level (ng·mL^−1^)							
*N*	28	3		7	16	8	
Mean (SD)	28.0 (64.22)	6.3 (7.33)	0.640	7.5 (5.17)	13.9 (34.10)	66.0 (105.31)	0.052
Timing of metastases, *N* (%)							
*N*	31	3		7	16	10	
Metachronous	11 (35.5)	1 (33.3)	1.000	2 (28.6)	7 (43.8)	3 (30.0)	0.715
Synchronous	20 (64.5)	2 (66.7)		5 (71.4)	9 (56.3)	7 (70.0)	
Sex, *N* (%)							
*N*	31	3		7	16	10	
Male	18 (58.1)	3 (100.0)	0.270	6 (85.7)	7 (43.8)	8 (80.0)	0.081
Female	13 (41.9)	0 (0.0)		1 (14.3)	9 (56.3)	2 (20.0)	
Tumor sidedness, *N* (%)							
*N*	30	3		7	16	10	
Right‐sided	6 (20.0)	1 (33.3)	0.524	1 (14.3)	3 (18.8)	3 (30.0)	0.742
Left‐sided (with rectum)	24 (80.0)	2 (66.7)		6 (85.7)	13 (81.3)	7 (70.0)	

### 3.4 Patient and biomarker characteristics according to neoadjuvant chemotherapy: the impact of neoadjuvant FOLFOX on immune infiltrate in metastases

Oxaliplatin‐based chemotherapy can release antigens and promote immunogenic cell death leading to a specific antitumor immune response. Therefore, we further examined the distribution and type of the immune cell infiltration in patients with or without neoadjuvant FOLFOX chemotherapy (Table 1, Table S3). Patients with chemotherapy‐treated metastatic sites had metastases strongly (IHC 3) positive for CD3 cells at IF (28.2% versus 14.7%), moderately (IHC 2)/strongly positive for CD3 cells in stroma (47.5% versus 32.4%), and moderately/strongly positive for CD8 cells at IF (41.1% versus 21.2%) than patients whose metastases were not exposed to chemotherapy. FOXP3 reg T‐cell staining was relatively weak but significantly decreased in the IF after chemotherapy (*P* = 0.0010). Of interest, seven chemo‐naive patients (out of 33; 21%) had high CD8 and high /PD‐L1 staining versus 13 who received FOLFOX‐based chemotherapy (out of 38; 34%).

These results suggested that chemotherapy may impact the immune microenvironment of patients and increase CD8 and PD‐L1 expression in metastases.

Finally, a significant inverse correlation between CD3^high^ T‐cell infiltration in stroma and TRG was observed, reflecting a better pathological response in CD3 T cells‐inflamed tumors (Spearman correlation −0.33, *P* = 0.0484; Table S4).

### 3.5 Association between the immune infiltrate and survival in metastases

**Fig. 2:**
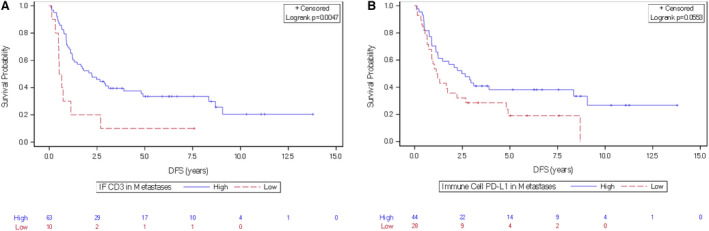
Kaplan–Meier curves showing the association between CD3^high^ T cell and PD‐L1 markers and DFS in patients with mCRC. (A) High and low CD3 in the invasive front at the metastatic site, (B) high and low PD‐L1 expression on immune cells at the metastatic site.

In the univariate analysis, low IF CD3 in metastases correlated with a shorter DFS (Table 3). Patients with a higher number of metastases and neoadjuvant chemotherapy had shorter DFS. The only variable identified as a prognostic factor for DFS in the multivariate analysis was IF CD3^high^ T‐cell infiltrate (HR = 0.31, 95% CI: 0.15–0.67, *P* = 0.002). DFS was significantly better in patients with high IF CD3 expression (IHC 2–3) than in those with low IF CD3 expression (median DFS of 2.2 years, 95% CI: 1.2–3.9 versus 0.59 years, 95% CI: 0.13–1.13, respectively; HR = 0.36, *P* = 0.005, Fig. 2A).

**Table 3:** Univariate and multivariate analyses for disease‐free survival. IC, immune cell; TC, tumor cell.

Parameter	Univariate analysis		Multivariate analysis	
	HR (95% CI)	*P*	HR (95% CI)	*P*
Age (years)	1 (0.970–1.029)	0.951		
Preoperative CEA level	1 (1.000–1.002)	0.130		
Chemotherapy				
No	1		1	
Neoadjuvant	1.99 (1.147–3.453)	**0.014**	1.682 (0.918–3.082)	0.093
Longest diameter of metastases (cm)				
≤ 5	1			
> 5	1.29 (0.604–2.749)	0.513		
N‐stage				
N0	1			
N+	1.38 (0.789–2.413)	0.259		
Number of metastases				
≤ 1	1		1	
> 1	2.12 (1.214–3.688)	**0.008**	1.816 (0.987–3.344)	0.055
Timing of metastases				
Metachronous	1			
Synchronous	0.93 (0.489–1.778)	0.832		
TRG				
2–3	1			
4–5	1.02 (0.465–2.256)	0.952		
Sex				
Male	1			
Female	1.41 (0.817, 2.451)	0.216		
Tumor sidedness				
Right‐sided	1			
Left‐sided (with rectum)	1.71 (0.728, 4.020)	0.218		
CD3+ IF				
Low	1		1	
High	0.36 (0.175–0.755)	**0.007**	0.309 (0.145–0.657)	**0.002**
CD3+ stroma				
Low	1			
High	1.19 (0.692–2.032)	0.534		
CD3+ IE				
Low	1			
High	0.71 (0.380–1.333)	0.288		
CD8+ IF				
Low	1			
High	1.21 (0.688–2.133)	0.507		
CD8+ stroma				
Low	1			
High	1.47 (0.776–2.787)	0.237		
PD‐L1 TC				
Low	1			
High	0.80 (0.286–2.213)	0.661		
PD‐L1 IC				
Low	1			
High	0.58 (0.335–1.020)	0.059		
CD8+ IF and PD‐L1 IC				
CD8low PD‐L1low	1			
CD8hi PD‐L1hi	0.79 (0.405–1.526)	0.477		
CD8hi PD‐L1low	1.21 (0.278–5.215)	0.803		
CD8low PD‐L1hi	0.48 (0.238–0.981)	**0.044**		
FOXP3 IF				
Staining 0	1			
Staining 1	0.66 (0.313–1.412)	0.288		
FOXP3 stroma				
Staining 0	1			
Staining 1	0.59 (0.263–1.301)	0.188		
Staining 2	0.57 (0.138–2.356)	0.437		
PD‐1 IF				
Low	1			
High	0.49 (0.192–1.264)	0.141		
PD‐1 stroma				
Low	1			
High	0.57 (0.138–2.351)	0.437		

Bold values indicate significance of *P* < 0.05.

A similar pattern of differential DFS according to the IF CD3^high^ score in metastases was observed when the analysis was restricted to patients with synchronous metastases (Fig. S4). Given the relatively small size of our cohort, we did not analyze IF CD3^high^ T‐cell expression impact on DFS in patients with metachronous metastases.

According to stratification based on PD‐L1 expression by immune cells in metastases, the median DFS was significantly better in patients with high PD‐L1 expression (the median DFS = 2.56 years, 95% CI: 1.11–8.34 versus 1.17 years, 95% CI: 0.73–2.22, respectively; HR = 0.58, 95% CI: 0.34–1.02, *P* = 0.05, Fig. 2B). The DFS was not significantly different regarding metastatic CD8^high^ expression in stroma or at the IF.

A schematic graphical summary of the key points is presented in Fig. S5.

## 4 Discussion

Assessment of tumor immune infiltrating cells is emerging as an important prognostic tool to stratify cancer patients according to the immune microenvironment. However, there are currently limited data on such analysis between the primary and paired metastatic tumor in patients with mCRC. In this study, we observed the intrapatient heterogeneity of immune infiltrates in both chemotherapy‐naive primary tumors and in the matched metastases (mainly in the liver) in 74 patients with mCRC.

Many studies explored immune infiltrate across tumorigenesis, with approaches outlining the difference in the immune contexture between primary and metastatic tumors [38, 39]. In the study by Angelova et al. [38], a thorough genomic and immunological characterization of primary and the matched metastatic tumor of two patients with CRC did not show any correlation, suggesting a high level of tumor heterogeneity between all lesions. This observation was further clearly confirmed by a Consensus Molecular Subtype (CMS) characterization in omCRC patients [39]. The authors showed that CMS subtypes, determined in patients undergoing partial hepatectomy of resectable CRC liver metastases, were variable. Gene expression analysis showed the absence of CMS1 (1%) and CMS3 (0%) subtypes in liver metastases and their presence in in the primary tumors (14% and 13%, respectively). Therefore, the immune profiling performed on the primary CRC has a limited predictive value to estimate the immune contexture in metastases.

In our analysis, the immune infiltrate in the primary tumor was not associated with survival (data not shown), except for stromal FOXP3. Previously published reports showed discordant results regarding the prognostic value of FOXP3 probably reflecting the plasticity of these immune subsets. Our results provide evidence that CD3 score in the IF of metastases has an independent prognostic value for DFS, doubling the survival rate, independent of TRG, further confirming the previously published data [1, 13, 40]. Contrary to Mlecnik et al. [2], we did not observe higher IF CD3 in patients with lower TRG (data not published). The median DFS was also significantly better in patients with higher immune PD‐L1 expression in metastases in our study. In another study, PD‐L1 expression in tissue microarray of surgically excised pMMR CRC patients was associated with improved overall survival, but analyses were performed only on primary tumors [20]. Considering these observations, it appears more pertinent to explore and characterize the immune infiltrate of metastases and to correlate it with patient prognosis for the stratification of patients for appropriate therapies.

An efficient immune response is characterized by activation of the interferon signaling pathway generating mature cytotoxic lymphocytes. PD‐L1 expression on tumor or immune cells is induced by an interferon‐mediated signaling and hence a subpopulation of patients with such expression seems to be of particular interest. In general, CD8^high^/PD‐L1 ^high^ cells were previously described as functional effector cells as they produce significantly higher level of Interferon Gamma (IFN‐γ) and express more the degranulation marker CD107a than CD8^low^/PD‐L1^low^ cells [41]. Although we did not assessed survival for the immune PD‐L1 and CD8^high^ groups due to the small number of patients in each group, the characterization of this subgroup was of importance in pMMR CRC patients. For the first time, we showed that this entity seems to be associated with lower tumoral mass and lower CEA. Lower stroma stiffness is one of the hypotheses explaining the enhancement of the CD8^high^/PD‐L1^high^ population, supported by recent observation correlating desmoplastic angiogenic stroma and CD8^high^ T‐cell immune infiltration in CRC liver metastasis [42]. This is in accordance with the previous observations by Wang et al. [40], showing the immune infiltrate associated with metastatic size, number of metastases, and Fong clinical Risk Score in resected CRC liver metastases.

The effect of chemotherapy on the CD8^high^/PD‐L1^high^ signature is of importance to better personalize treatment strategies. In our cohort, we observed significantly more patients harboring CD8^high^/PD‐L1^high^ staining in the group exposed to neoadjuvant FOLFOX‐based chemotherapy. The T lymphocyte density and location in metastatic melanomas were reported to have predictive value for treatment outcome of patients receiving anti‐PD‐1/PD‐L1 therapies [43]. Although an increase in CD8^high^ and PD‐L1 immune infiltrate by chemotherapy did not significantly correlate with survival, we hypothesize that this specific subgroup of patients as identified herein may benefit from personalized treatment including ICIs. Effect of chemotherapy on the immune infiltrate was described in nonmetastatic rectal cancer, with an increased stromal CD8^high^ and CD4 T cells after chemoradiotherapy, associated with a better prognosis [44]. Recently, the pooled analysis of postchemotherapy resected metastases of pMMR CRC confirmed the good prognosis of patients with such an inflamed microenvironment [23]. However, this analysis did not include paired tumors before and after treatment. Using a combination of chemotherapies with ICIs may improve response rate, as recent data showed up to 27% of pathological response in pMMR CRC patients after nivolumab and ipilimumab neoadjuvant therapy [19]. The tissue immune profiling could allow design of immunotherapies for the CD8^high^/PD‐L1^high^ subset of patients in the adjuvant setting of metastasectomy. It could also help to plan strategies in the following chemo‐immunotherapy lines of treatment. This is consistent with the recent results reported by Kumagai et al. [45], showing that a profound reactivation of effector PD‐1^+^CD8^+^ T cells is necessary for tumor regression, which paves the way for a promising predictive biomarker for PD‐1 blockade therapies.

Our study has several limitations. Firstly, the immune infiltrate was investigated in only one metastatic lesion per patient. A major obstacle to a refined definition of the immune contexture of human CRC liver metastases resides in the heterogeneity between the different metastases in the same patient, in addition to the profound heterogeneity of tumor lesions across patients. Galon et al. demonstrated that the immune phenotype of the least‐infiltrated metastasis had a stronger association with patient outcome than other metastases [6]. This effort of selection could not be performed here. Nevertheless, in our study, IHC analyses were performed with the semiquantitative method on the entire slide within three separate compartments of the tumor. Secondly, this study does not include *RAS* mutational status. It was reported that CRC harboring *RAS* mutations or mitogen‐activated protein kinase kinase (MEK) activation had less major histocompatibility complex‐I (MHC‐I) expression and lower CD8 T‐cell activation [46]. Neoadjuvant chemotherapy was previously shown to enhance CD8 and immune PD‐L1 expression in metastases of *RAS* wild‐type cancers [47], and this will be crucial to analyze in validation cohorts. Finally, the peripheral immune response could not be assessed in complement to the intratumoral approach. The initiation of cell death and subsequent activation of T cells when antigens are released can be monitored by different methods. The peripheral immune response against tumor antigens before and after administration of the FOLFOX regimen has been previously assessed in mCRC patients [48]. An epitope spreading stimulating immune response against a broad spectrum of tumor antigens hinders monitoring the effect of immune‐based chemotherapy in the circulating blood of patients. Peripheral blood leucocytes phenotypic profiling is easy to perform and decreased levels of myeloid‐derived suppressor cells (MDSC) and increased levels of circulating CD8+ T cells lymphocytes after 5‐fluorouracil‐based chemotherapy have been detected by flow cytometric analyzes [49]. Doublet chemotherapies with the FOLFOX +/− bevacizumab induce a significant decrease in the number of MDSC, specifically granulocytic MDSC, which was associated with better progression‐free survival in patients receiving this combination [50, 51]. A combined peripheral and intratumoral assessment of the immune response is a promising approach for the future studies.

## 5 Conclusion

In conclusion, our findings suggest an effect of chemotherapy on modifying the immune microenvironment in resected CRC metastases and highlight the relevance of CD8^high^/PD‐L1^high^ in pMMR/MSS metastases for adjuvant treatments including immunotherapy strategies. This key immune signature based on the assessment of CD8 and PD‐L1 by IHC, adapted for a routine practice and a cost‐effective method, paves the way for further prospective analyses in mCRC and encourages the efforts for such rare data sharing.

## Conflict of interest

The authors declare no conflict of interest.

## Author contributions

MJ involved in formal analysis and validation, and wrote original draft. W‐WL performed statistical analysis and critically reviewed the manuscript. DY performed formal analysis and critically reviewed the manuscript. IB performed statistical analysis and critically reviewed the manuscript. AM, EC, AT, RC, and DV critically reviewed the manuscript. IB performed data curation and critically reviewed the manuscript. NR‐R, PB, and FP‐L involved in complementary investigation and critically reviewed the manuscript. TA involved in conceptualization, formal analysis, validation, and investigation and critically reviewed the manuscript. CB and KS involved in formal analysis and validation, and critically reviewed the manuscript. MS involved in conceptualization, data curation, formal analysis, validation, and investigation and wrote original draft.

### Peer Review

The peer review history for this article is available at https://publons.com/publon/10.1002/1878‐0261.13173.

## Supporting information


**Fig. S1.** Flow chart of the study.

**Fig. S2.** Heatmaps of biomarkers expression in the primary and metastatic tumor.

**Fig. S3.** Representative images of immunohistochemistry co‐staining of CD8 and PD‐L1.

**Fig. S4.** Kaplan‐Meier curve showing the association between CD3 ^high^ in the invasive front and DFS in mCRC patients with synchronous metastases.

**Fig. S5.** Graphical summary of the results.

**Table S1.** Raw data.

**Table S2.** The cohort baseline characteristics.
**Table S3.** Patients' and metastatic biomarkers characteristics according to neoadjuvant or adjuvant chemotherapy.
**Table S4.** Spearman correlation between metastatic immune infiltrate and TRG scoring.

## Data Availability

The datasets used and/or analyzed during the present study are available within the supplementary figures and tables.
