# Supplementary figures and images for: Immune microenvironment in patients with mismatch‐repair‐proficient oligometastatic colorectal cancer exposed to chemotherapy: the randomized MIROX GERCOR cohort study
Source: Mol Oncol. 2022 Feb 9;16(11):2260–73. doi: 10.1002/1878-0261.13173 (PMC9168761; doi:10.1002/1878-0261.13173)

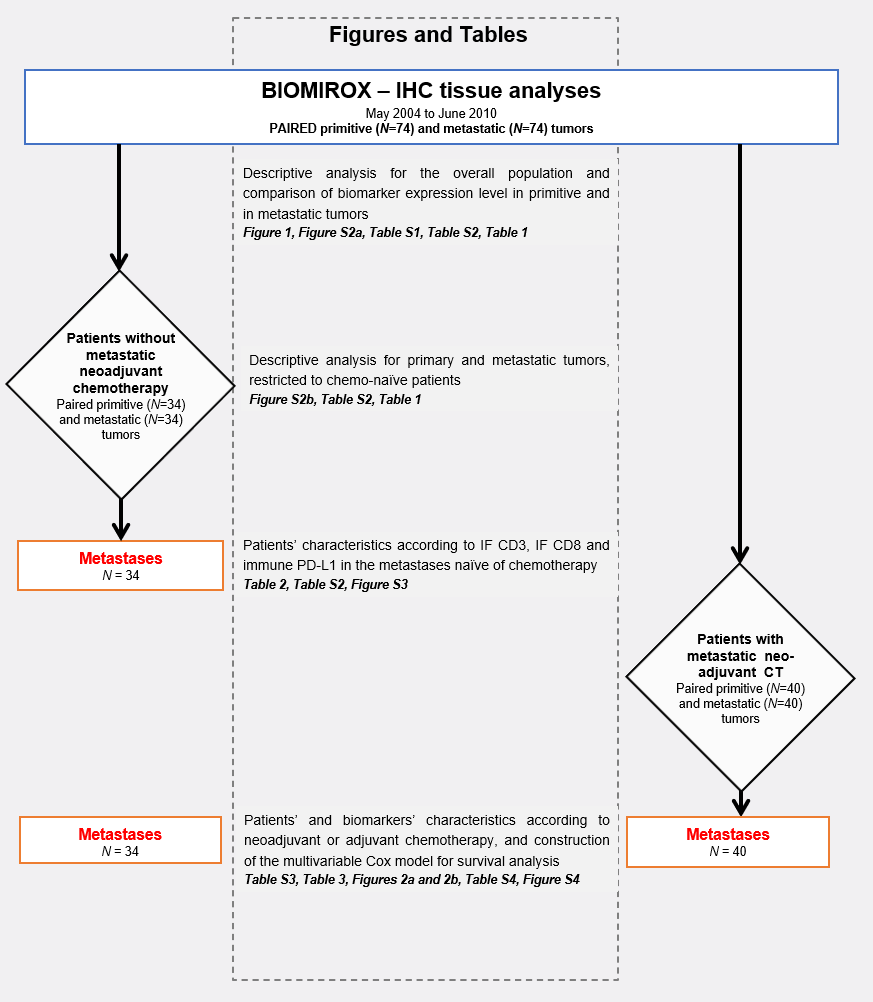

Supplement: Supplementary file 1 — Fig. S1. Flow chart of the study. [file MOL2-16-2260-s007.tif]

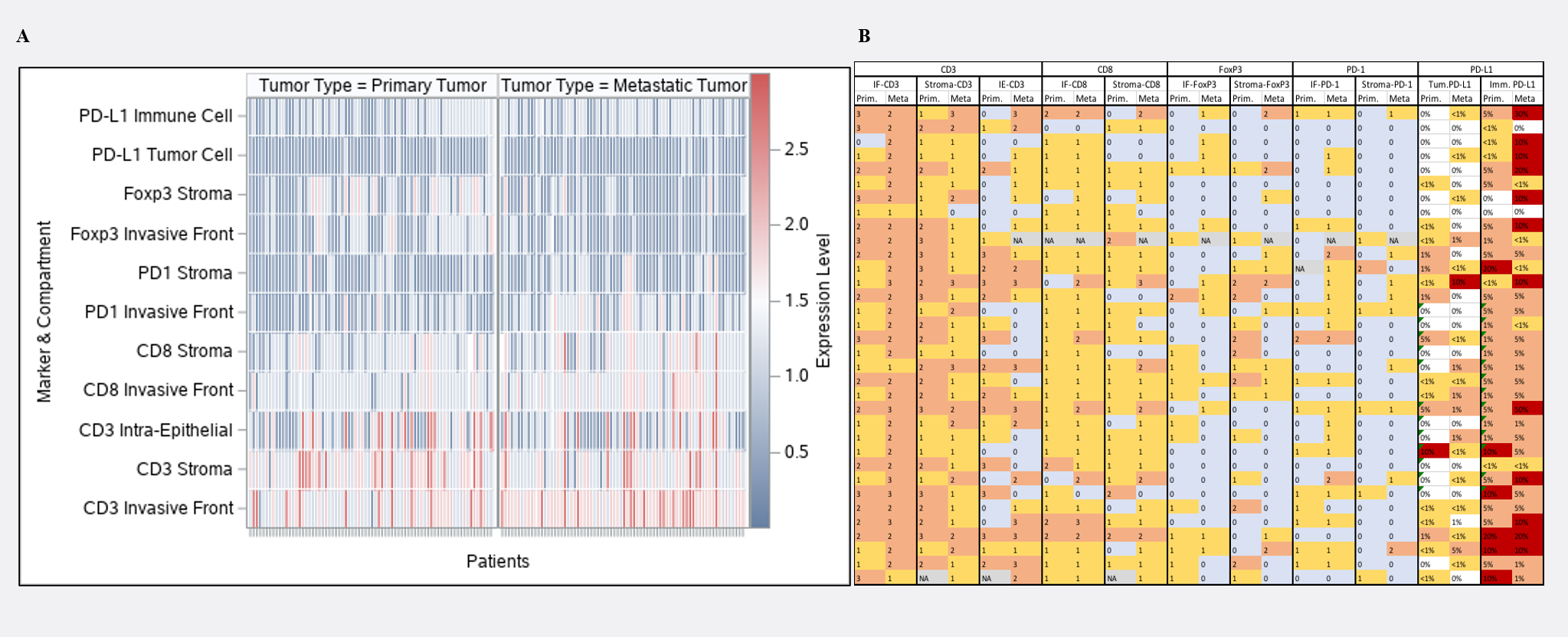

Supplement: Supplementary file 2 — Fig. S2. Heatmaps of biomarkers expression in the primary and metastatic tumor. [file MOL2-16-2260-s002.tif]

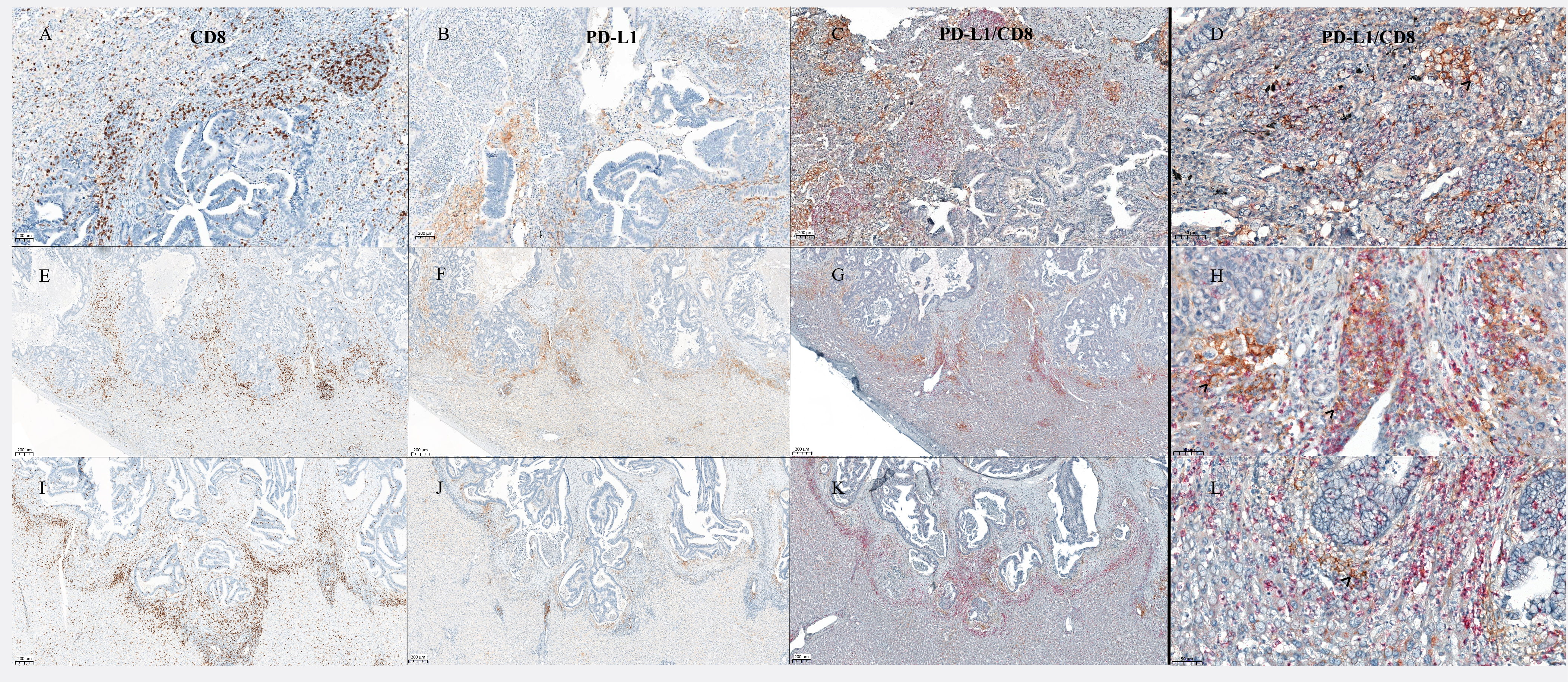

Supplement: Supplementary file 3 — Fig. S3. Representative images of immunohistochemistry co‐staining of CD8 and PD‐L1. [file MOL2-16-2260-s006.tif]

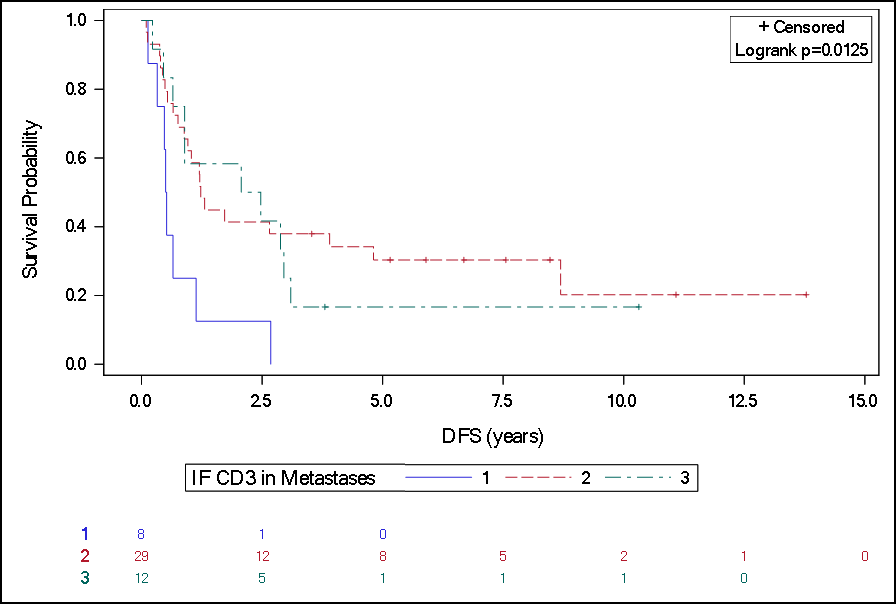

Supplement: Supplementary file 4 — Fig. S4. Kaplan‐Meier curve showing the association between CD3 high in the invasive front and DFS in mCRC patients with synchronous metastases. [file MOL2-16-2260-s003.tif]

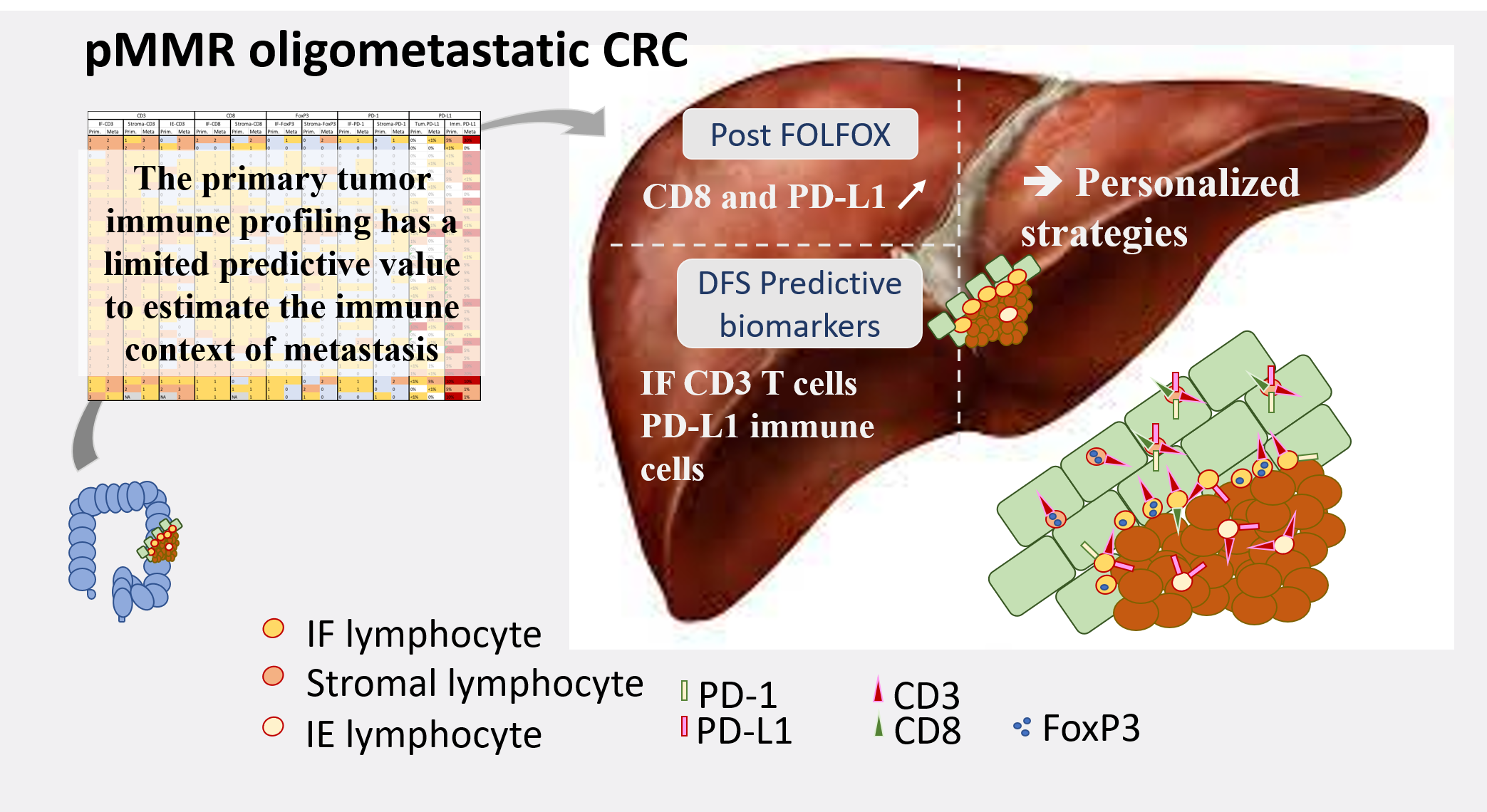

Supplement: Supplementary file 5 — Fig. S5. Graphical summary of the results. [file MOL2-16-2260-s005.tif]
