# Supplementary material for: Immune microenvironment in patients with mismatch‐repair‐proficient oligometastatic colorectal cancer exposed to chemotherapy: the randomized MIROX GERCOR cohort study
Source: Mol Oncol. 2022 Feb 9;16(11):2260–73. doi: 10.1002/1878-0261.13173 (PMC9168761; doi:10.1002/1878-0261.13173)
Supplement: Supplementary file 7 — Table S2. The cohort baseline characteristics. Table S3. Patients' and metastatic biomarkers characteristics according to neoadjuvant or adjuvant chemotherapy. Table S4. Spearman correlation between metastatic immune infiltrate and TRG scoring. [file MOL2-16-2260-s004.docx]

**The immune microenvironment in patients with mismatch-repair-proficient oligometastatic colorectal cancer exposed to chemotherapy: the randomized MIROX GERCOR cohort study**

**Supplementary tables (excluding Table S1) and legends**

**Table S1. Raw data**

See separate data table

**Table S2. The cohort baseline characteristics**

| **Parameter** |  | **Overall** |
| --- | --- | --- |
| **Age (years)**  ***N* = 74** | **Median (range)** | **61.4 (29-75)** |
| **MMR status, *N* (%)**  ***N* = 74** | pMMR | 74 (100) |
|  | dMMR | 0 (0.0) |
| **Timing of metastases, *N* (%)**  ***N* = 74** | Metachronous | 24 (32.4) |
|  | Synchronous | 50 (67.6) |
| **Chemotherapy status at metastatic surgery time, *N* (%)**  ***N* = 74** | Neoadjuvant, all chemotherapies | 40 (54.1) |
|  | Oxaliplatin-based neoadjuvant | 38 (51.4) |
|  | Chemo-naïve | 34 (45.9) |
| **Longest diameter of metastases, *N* (%)**  **(cm)**  ***N* = 72** | <=5 | 63 (87.5) |
|  | >5 | 9 (12.5) |
| **N-stage, *N* (%)**  ***N* = 72** | N+ | 41 (56.9) |
|  | N0 | 31 (43.1) |
| **Number of metastases, *N* (%)**  ***N* = 72** | Median | 2.0 |
|  | 1 | 33 (45.8) |
|  | 2 | 19 (26.4) |
|  | 3 | 8 (11.1) |
|  | 4 | 7 (9.7) |
|  | 5 | 2 (2.8) |
|  | 6 | 2 (2.8) |
|  | 7 | 1 (1.4) |
| **Site of metastases, *N* (%)**  ***N* = 74** | Liver | 65 (87.8) |
|  | Lung | 3 (4.0) |
|  | Ovary | 1 (1.4) |
|  | Peritoneum | 5 (6.8) |
| **Preoperative CEA level (ng/mL)**  ***N* = 70** | Mean (SD) | 55.9 (209.36) |
| **TRG scoring, *N* (%)**  ***N* = 36** | 2-3 | 12 (33.3) |
|  | 4-5 | 24 (66.7) |
| **Sex, *N* (%)**  **N = 74** | Male | 48 (64.9) |
|  | Female | 26 (35.1) |
| **Tumor Sidedness, N (%)**  ***N* = 69** | Right-Sided | 12 (17.4) |
|  | Left-Sided (with Rectum) | 57 (82.6) |

**Abbreviations:** pMMR, proficient mismatch repair; dMMR, deficient mismatch repair; TRG, tumor regression grades

**Table S3.** **Patients’ and metastatic biomarkers characteristics according to neoadjuvant or adjuvant chemotherapy**

| **Parameter** |  | **Adjuvant** | **Neoadjuvant** | | ***P*** | |  |
| --- | --- | --- | --- | --- | --- | --- | --- |
| **Age (years)** | *N* | 34 | 40 | |  | |  |
|  | Median (range) | 62 (45-75) | 61.1 (29-75) | | 0.323 | |  |
| **Longest diameter of metastases (cm), *N* (%)** | <=5 | 31 (93.9) | 32 (82.1) | | 0.166 | |  |
|  | >5 | 2 (6.1) | 7 (17.9) | |  | |  |
|  |  |  |  | |  | |  |
| **N-stage, *N* (%)** | N0 | 18 (54.5) | 13 (33.3) | | 0.096 | |  |
|  | N+ | 15 (45.5) | 26 (66.7) | |  | |  |
|  | missing | 1 | 1 | |  | |  |
| **Number of metastases** | Mean (SD) | 1.6 (1.22) | | 2.5 (1.45) | | 0.001 | |
|  | missing | 1 | | 0 | |  | |
| **Number of metastases, *N* (%)** | <=1 | 22 (66.7) | | 12 (30.0) | | 0.002 | |
|  | >1 | 11 (33.3) | | 28 (70.0) | |  | |
|  | missing | 1 | | 0 | |  | |
| **Preoperative CEA level (ng/mL)** | Mean (SD) | 25.9 (61.31) | | 79.8 (274.41) | | 0.187 | |
|  | missing | 3 | | 1 | |  | |
| **Timing of metastases, *N* (%)** | Metachronous | 12 (35.3) | | 2 (5.0) | | 0.002 | |
|  | Synchronous | 22 (64.7) | | 38 (95.0) | |  | |
|  | missing | 0 | | 0 | |  | |
| **Sex, *N* (%)** | Male | 21 (61.8) | | 27 (67.5) | | 0.633 | |
|  | Female | 13 (38.2) | | 13 (32.5) | |  | |
|  | missing | 0 | | 0 | |  | |
| **Tumor Sidedness, N (%)** | Right-Sided | 7 (21.2) | | 5 (13.9) | | 0.531 | |
|  | Left-Sided (with Rectum) | 26 (78.8) | | 31 (86.1) | |  | |
|  | missing | 1 | | 4 | |  | |
| **CD3+ IE, *N* (%)** | Low | 21 (63.6) | | 32 (80.0) | | 0.187 | |
|  | High | 12 (36.4) | | 8 (20.0) | |  | |
|  | missing | 1 | | 0 | |  | |
| **CD3+ IF, *N* (%)** | Low | 3 (8.8) | | 7 (17.9) | | 0.321 | |
|  | High | 31 (91.2) | | 32 (82.1) | |  | |
|  | missing | 0 | | 1 | |  | |
| **CD3+ Stroma, *N* (%)** | Low | 23 (67.6) | | 21 (52.5) | | 0.237 | |
|  | High | 11 (32.4) | | 19 (47.5) | |  | |
|  | missing | 0 | | 0 | |  | |
| **CD8+ IF, *N* (%)** | Low | 26 (78.8) | | 23 (59.0) | | 0.083 | |
|  | High | 7 (21.2) | | 16 (41.0) | |  | |
|  | missing | 1 | | 1 | |  | |
| **CD8+ Stroma, *N* (%)** | Low | 27 (81.8) | | 29 (74.4) | | 0.573 | |
|  | High | 6 (18.2) | | 10 (25.6) | |  | |
|  | missing | 1 | | 1 | |  | |
| **CD8+ IF and PD-L1 IC, *N* (%)** | CD8high PD-L1high | 7 (21.2) | | 13 (34.2) | | 0.061 | |
|  | CD8high PD-L1low | 0 (0.0) | | 2 (5.3) | |  | |
|  | CD8low PD-L1high | 16 (48.5) | | 8 (21.1) | |  | |
|  | CD8low PD-L1low | 10 (30.3) | | 15 (39.5) | |  | |
|  | missing | 1 | | 2 | |  | |
| **FOXP3 IF, *N* (%)** | Staining 0 | 21 (63.6) | | 37 (94.9) | | 0.001 | |
|  | Staining 1 | 12 (36.4) | | 2 (5.1) | |  | |
|  | missing | 1 | | 1 | |  | |
| **FOXP3 Stroma, *N* (%)** | Staining 0 | 21 (63.6) | | 34 (87.2) | | 0.022 | |
|  | Staining 1 | 8 (24.2) | | 5 (12.8) | |  | |
|  | Staining 2 | 4 (12.1) | | 0 (0.0) | |  | |
|  | missing | 1 | | 1 | |  | |
| **PD1 IF, *N* (%)** | Low | 30 (90.9) | | 32 (84.2) | | 0.489 | |
|  | High | 3 (9.1) | | 6 (15.8) | |  | |
|  | missing | 1 | | 2 | |  | |
| **PD1 Stroma, *N* (%)** | Low | 32 (97.0) | | 35 (92.1) | | 0.618 | |
|  | High | 1 (3.0) | | 3 (7.9) | |  | |
|  | missing | 1 | | 2 | |  | |

**Abbreviations:** IF, invasive front; IE, intra-epithelial; IC, immune cell; CT, tumor cell

**Table S4: Spearman correlation between metastatic immune infiltrate and TRG scoring**

|  | ***N*** | | **Sample Correlation** | **95% Confidence Limits** | | ***P*** |
| --- | --- | --- | --- | --- | --- | --- |
| **CD3+ IE** | | 36 | -0.16203 | -0.463954 | 0.178114 | 0.348 |
| **CD3+ IF** | | 36 | -0.12682 | -0.435674 | 0.212218 | 0.464 |
| **CD3+ Stroma** | | 36 | -0.33064 | -0.591524 | 0.002365 | 0.048 |
| **CD8+ IF** | | 36 | -0.27483 | -0.550670 | 0.062947 | 0.105 |
| **CD8+ Stroma** | | 36 | -0.28663 | -0.559415 | 0.050348 | 0.090 |
| **FOXP3 IF** | | 36 | -0.13771 | -0.444484 | 0.201760 | 0.426 |
| **FOXP3 Stroma** | | 36 | -0.20848 | -0.500362 | 0.131812 | 0.224 |
| **PD1 IF** | | 35 | -0.12293 | -0.436765 | 0.221018 | 0.485 |
| **PD1 Stroma** | | 35 | -0.15934 | -0.465913 | 0.185926 | 0.363 |
| **PD-L1 IC** | | 35 | 0.01174 | -0.322921 | 0.343493 | 0.947 |
| **PD-L1 TC** | | 35 | -0.14187 | -0.452010 | 0.202875 | 0.419 |

**Abbreviations:** IF, invasive front; IE, intra-epithelial; IC, immune cell; CT, tumor cell

**Supplementary Figure Legends**

**Figure S1: Flow chart of the study**

**Figure S2: Heatmaps of biomarkers expression in the primary and metastatic tumor** (A) The whole cohort (*N* = 74) (B) Chemo-naive subgroup (*N* = 34). PD-L1 expression was categorized into high (1) and low (0) based on the 5% expression cutoff.

**Figure S3: Representative images of immunohistochemistry co-staining of CD8 and PD-L1.**

(A, B, C) Single CD8 (A) and PD-L1 (B) immunostainings and double-antibody immunohistochemistry CD8/PD-L1 (C) in 3 different cases at the invasive front of lung metastasis, (E,F,G, I,J, K) and at the invasive front of liver metastases (CD8 (E,I), PD-L1 (F,J), co-staining (G,K)).

At higher magnification (D, H and L), by double-antibody IHC, it appears that some CD8 positive cells (red chromogen) co-express PD-L1 (brown chromogen) (arrows).

*Scale bar for S3A, S3B, S3C, S3E, S3F, S3G, S3I, S3J, S3K = 200 µm.*

*Scale bar for S3D, S3H, S3L = 50 µm.*

**Figure S4: Kaplan-Meier curve showing the association between CD3 high in the invasive front and DFS in mCRC patients with synchronous metastases.**

*CI, confidence interval*

**Figure S5: Graphical summary of the results**
